# Worker Perspectives on Incorporating Artificial Intelligence into Office Workspaces: Implications for the Future of Office Work

**DOI:** 10.3390/ijerph18041690

**Published:** 2021-02-10

**Authors:** Yoko E. Fukumura, Julie McLaughlin Gray, Gale M. Lucas, Burcin Becerik-Gerber, Shawn C. Roll

**Affiliations:** 1 Chan Division of Occupational Science and Occupational Therapy, University of Southern California, Los Angeles, CA 90089, USA; fukumura@usc.edu (Y.E.F.); jmgray@usc.edu (J.M.G.); 2 Institute for Creative Technologies, University of Southern California, Los Angeles, CA 90089, USA; lucas@ict.usc.edu; 3 Sonny Astani Department of Civil and Environmental Engineering, University of Southern California, Los Angeles, CA 90089, USA

**Keywords:** workspace, office work, computer workstations, artificial intelligence

## Abstract

Workplace environments have a significant impact on worker performance, health, and well-being. With machine learning capabilities, artificial intelligence (AI) can be developed to automate individualized adjustments to work environments (e.g., lighting, temperature) and to facilitate healthier worker behaviors (e.g., posture). Worker perspectives on incorporating AI into office workspaces are largely unexplored. Thus, the purpose of this study was to explore office workers’ views on including AI in their office workspace. Six focus group interviews with a total of 45 participants were conducted. Interview questions were designed to generate discussion on benefits, challenges, and pragmatic considerations for incorporating AI into office settings. Sessions were audio-recorded, transcribed, and analyzed using an iterative approach. Two primary constructs emerged. First, participants shared perspectives related to preferences and concerns regarding communication and interactions with the technology. Second, numerous conversations highlighted the dualistic nature of a system that collects large amounts of data; that is, the potential benefits for behavior change to improve health and the pitfalls of trust and privacy. Across both constructs, there was an overarching discussion related to the intersections of AI with the complexity of work performance. Numerous thoughts were shared relative to future AI solutions that could enhance the office workplace. This study’s findings indicate that the acceptability of AI in the workplace is complex and dependent upon the benefits outweighing the potential detriments. Office worker needs are complex and diverse, and AI systems should aim to accommodate individual needs.

## 1. Introduction

Most office workspaces do not adequately support office worker health, nor do they maximize work performance. Indoor environmental factors, such as temperature, air quality, and lighting, which do not fit the individual’s needs or support occupational requirements, can directly affect office workers [1,2,3]. In the U.S., most heating, ventilation, and air conditioning (HVAC) systems are centrally controlled within a narrow range; exposure to greater temperature variability may support improved health (e.g., glucose handling, cardiovascular performance) [4]. Moreover, offices maintained at thermal levels that are too cold or too warm can negatively impact productivity and require workers to exert more effort to complete tasks [5]. In addition to temperature, lighting can negatively impact alertness, mood, and performance [6], cause eye strain and lead to problems with visual acuity [7], and disrupt an individual’s natural circadian rhythm [8]. Finally, ambient noise, such as that from office equipment and coworkers talking, can be counterproductive, cause increased frustration, and lead to declines in worker well-being [9,10]. 

In addition to direct effects of the workplace physical environment, these and a variety of other factors intersect to affect worker health and well-being in office workers. Poor lighting, temperature, and other environmental factors can lead to maladaptive postures by causing an individual to hunch or move closer to the task [11], further exacerbating one of the most common health issues in office workers, musculoskeletal disorders. In fact, musculoskeletal conditions are the most commonly referenced health problems, with an annual prevalence of musculoskeletal pain in as many as 63% of office workers [12]. Factors such as job satisfaction and affective well-being can play a mediating role between the physical work environment and a worker’s absenteeism or presenteeism, a relationship that is further impacted by an individual’s work patterns [13]. Among all of the individual work patterns, the most common concern is that office workers spend an average of 11.6 h of their day in sedentary activity [14], which is directly associated with numerous adverse health impacts, including musculoskeletal disorders and metabolic syndromes [15,16,17]. Beyond detriments to health, sedentary behavior has been associated with lower cognitive performance [18,19]. 

Fluctuations in social and organizational practices, changes in worker demographics, and advances in technology are rapidly reshaping the future of office work in ways that may amplify these potential detriments to worker health and well-being [20]. While the contemporary trend in offices was to offer open environments with shared workstations (e.g., hot-desking) [21,22], the COVID-19 pandemic has recently led organizations to consider alternative workflows and work arrangements [23,24]. Of note is an increasing use of digital technologies to support remote work that has led to an increase in computer workstation use, with workers spending up to 1.5 h per day longer at their workstations than when working in-person [25]. Future work design that includes higher frequency of remote work creates additional risks to worker health and well-being. These factors go beyond the physical workspace and include social and personal factors such as the frequency of communication with co-workers and interruptions from other individuals in the home workspace [25]. 

The plethora of health and performance issues associated with office work are likely to continue and expand in future work and workplaces [21,26]; thus, it is ever important to identify solutions to the challenges of mitigating risk and improving well-being within office work [27]. Environmental modifications such as sit-stand desks have been found to reduce sedentary work behavior in the short term, but no studies examining these modifications have demonstrated long-term effectiveness of reducing sedentary behavior [28] nor any effects in reducing pain-related disability in office workers [29]. Workplace wellness programs have also been developed to address health and performance issues; however, retention of positive change is difficult, and predictors of successful intervention are not yet well-understood [30,31]. Such interventions are difficult because the workplace environment, the individual, and other contextual factors all affect engagement in preventive behaviors in the workplace [32]. Furthermore, changing workplace behavior requires altering habits that people have developed in their workspaces over time. Once formed, habits can dictate how a person engages with their environment, even when those behaviors have negative health impacts [33]. These factors are further amplified by the ever-increasing diversity in the workforce, work design, and work settings, and there is a growing need for a multifaceted approach that can support worker health and well-being [20,34]. 

Artificial intelligence (AI) may be a viable solution for delivering such multifaceted large scale, yet individualized, interventions to address environmental conditions and worker behaviors to promote healthy, supportive office workspaces. Automation in the workplace has been primarily focused on incorporating machines in service-oriented industries or jobs requiring manual labor to supplement or replace the actions of human workers [35]; however, some autonomous support systems are being developed to support worker health, such as to encourage exercise, or to support worker productivity, such as in decision-making tasks [36,37,38]. Unfortunately, most of these systems tend to take workers away from their work tasks to provide information or communicate with the system [39]. There is a growing need to develop AI systems that can support worker health and productivity without interrupting job performance. Moreover, despite the increasing prevalence of automated systems in the workplace, there remains speculation and varying levels of acceptance of AI due to factors such as fear of the AI [40] and privacy concerns [41].

There is a clear opportunity for integrating an AI system that can learn individual needs, improve the work environment, and support behavior change over time within office workspaces. We previously completed and published a comprehensive review of current evidence and gaps in knowledge relative to developing an AI system for this purpose [42], through which we determined that it is necessary to begin by understanding office workers’ perspectives, needs, and preferences. Specifically, there is limited knowledge of how the implementation of AI would alter the occupational context for workers in an office setting and how to develop an acceptable AI system that could become fully incorporated without being obtrusive. To examine these concepts, we conducted focus group interviews to explore office workers’ perspectives on introducing AI into their workspace. In these focus groups, participants were not reacting to or providing feedback on a specific AI system; rather, these discussions were used to generate ideas and better understand how to design an AI system that would be most effective at achieving outcomes of importance to office workers such that the design would be acceptable to end-users.

## 2. Materials and Methods

### 2.1. Framework of the Study

The long-term goal of our research is to develop an AI-enabled office workstation that can support the health and well-being needs of office workers. We will do so by integrating sensors and feedback systems into the office environment that will enable the worker and AI to work synergistically [42]. At the outset of this broader project, we engaged with office workers to identify their perspectives so that we might design a system that could be easily translated into practice for maximum effects on the intended outcomes. Thus, we implemented a descriptive qualitative approach using focus group interviews with office workers. Qualitative approaches allow for deeper exploration of individual and group perspectives on a topic of interest than cannot be gained through quantitative means [43]. AI in the workspace can be a foreign concept for office workers who have become accustomed to their traditional workplace environments. A focus group method was chosen to gain a deeper understanding of worker preferences regarding AI in their workplace environments that cannot be as easily gathered through quantitative or survey methods. Although not guaranteed, successful focus groups can capitalize on group dynamics and lead to rich discussions [44]. Furthermore, focus groups may allow for richer understanding by mitigating the researcher’s authority and giving space to the participants [45]. Focus group interviews were conducted in person or through video conferencing to enable as much interaction in real time and space as possible. The University of Southern California’s institutional review board approved the protocols and procedures (Study ID: UP-18-00131). The study was conducted in accordance with the Declaration of Helsinki, meeting both the U.S. and European Union requirements for human subject research. All participants provided informed consent to participate in the focus groups, including permission to be audio-recorded.

### 2.2. Participants

Six focus group interviews were conducted between January 2019 and May 2019 We recruited participants through network sampling, where our research team asked office workers within their professional circles to volunteer their time. A total of 45 office workers participated. Three group interviews were conducted with a total of 27 employees of Arup Group Ltd., an independent architecture and design firm specializing in complex built environments. Participants from Arup identified their occupations as engineers, architects, and other building consultants. Three additional group interviews were conducted with a total of 18 staff members from varying departments of University of Southern California (USC). These university participants were not faculty with teaching roles; rather, participants described having roles in management, administration, finance, and office assistant positions. There were more males in the Arup groups and more females in the university interviews (10 females and 17 males, 14 females and 4 males, respectively). While all participants reported spending the majority of their workday at desks, there was a range of office arrangements. Many engineers reported having an open office space with hot-desking, that is, the ability to work at a workstation of their choice, and a few participants reported having sit-to-stand desks. Three engineers and three university staff reported splitting their time between different office spaces (i.e., work from home). 

### 2.3. Focus Group Procedures

Of the six focus groups, four occurred in-person with employees from either USC or Arup working in Los Angeles, and two were conducted using video conferencing with Arup employees working at the company’s primary offices in London. During the focus groups, participants were asked open-ended questions related to integrating AI within their office workspace. An interview guide facilitated these semi-structured interviews and ensured that each group interview covered the same topics (Appendix A). Specifically, instead of feedback on an existing AI system, we framed these discussions around the proposed development of a new AI-enabled, smart office workstation that could provide support for improving health and work performance through automated feedback and environmental modifications [42]. At the start of each focus group, the moderator provided the following prompt to set the foundation for group discussion:

“We are looking at opportunities around making the work environment healthier for people. For example, there might be an artificial intelligence (AI) system that asks you to do healthier things like change the office temperature, change the room lighting, improve your posture, or change your position from sit to stand. We will use this data to inform the development of such technology.”

Following this introduction, participants were asked to imagine that AI was integrated into their office workspace to support their work performance and individual health. Participants were asked how they would like the AI to make changes or preferences related to receiving feedback from an AI workspace that could adjust the office environment (i.e., temperature and lighting), as well as the workstation itself (i.e., sitting or standing desk). This conversation was followed by questions that aimed to understand the worker’s level of comfort and preferences regarding sensors or other means for obtaining data that could be used by a smart workstation to monitor the worker’s health and the environment. At the end of each focus group, moderators asked participants what other options they would like in a “dream workstation.” Throughout the session, multiple follow-up prompts were used to probe for information on specific concepts and topics within the feedback, sensing, and additional features segments.

Each focus group interview was scheduled for 60 min, and the sessions lasted between 41 and 75 min. After each focus group interview, the research team discussed initial findings and modified the interview guide to pursue emerging topics not outlined in the original interview guideline. All interviews were audio recorded and later transcribed to text. Each transcript was coded with the location and employer (i.e., Arup or USC), and individual participant identities were blinded in the transcripts by coding each comment with a participant’s sex.

### 2.4. Data Analysis

Analysis of the focus group transcripts was completed by two researchers who were not involved in the interview guide’s initial development and did not moderate the interviews. Basic qualitative thematic analysis was conducted using an inductive data coding process using a systematic, four-step text condensation procedure [46]:

(1) Total impression—from chaos to themes: During an initial reading, the smallest meaning units were identified and notated with minimal interpretation. Two researchers independently read all six interviews after the initial calibration of notation.
(2) Identifying and sorting meaning units—from themes to codes: The two researchers discussed preliminary impressions and findings from the initial coding process, and they developed categorical codes through an iterative process.
(3) Condensation—from code to meaning: Both researchers coded all six interview transcripts using the developed categories. This coding process involved a constant comparative method where the two researchers modified the codes according to the transcript data and discussions and questions that arose during the process [47].
(4) Synthesizing—from condensation to descriptions and concepts: Using the coded transcripts, the two researchers identified themes and patterns in the data. Prominent themes relevant to the research questions were revisited and summarized to produce the final results and interpretation.

The researchers who moderated focus group sessions were consulted between the initial code development and finalization of the codes to ensure the credibility of the independent coders. Based on the team’s feedback, the independent coders then developed final themes and conducted an initial interpretation of the data. The full research team reviewed the interpretation and provided final feedback to ensure the accuracy, credibility, and trustworthiness of the findings.

### 2.5. Researcher Bias and Assumptions

The focus group moderators were interdisciplinary researchers engaged in developing AI solutions for office settings, including experts in human-computer interaction, engineering and design, and occupational science and ergonomics. Two investigators who did not engage in the focus group interviews conducted the qualitative review of the transcripts to minimize bias. One analyst is an occupational therapist and an occupational science PhD student research assistant affiliated with the broader study that funded this project but had not been involved in any aspect of the focus group interviews before reading the transcripts. To further increase the trustworthiness and rigor of the analysis, the second analyst was an outsider to the research project and an expert in qualitative methodology. She is an occupational scientist and occupational therapist who has written extensively on dynamic systems and occupation.

## 3. Results

We examined differences across groups to see if comments varied among those who worked at Arup versus USC, between groups conducted in London versus Los Angeles, or between participants of different sexes. We did not identify any significant differences in the discussions across groups conducted with engineering or university workers, between the two different geographic locations, or among workers with different job types or sexes. Two constructs emerged from the transcripts. First, participants shared perspectives related to their preferences and concerns regarding communication and interactions with AI technology within an office setting. Second, numerous conversations highlighted the dualistic nature of a system that collects large amounts of data; that is, the potential benefits for behavior change to improve health and the perils of trust and privacy. Further, across both constructs, there was an overarching discussion related to the complexity of work performance and its implications for incorporating AI into office settings. Finally, participants shared numerous thoughts relative to future AI solutions that could enhance the office workplace. Rich discussions revealed the need to individualize AI workstations to various user preferences and how the acceptability of an AI workstation is complex and affected by the person, context, and individual occupations. 

### 3.1. Communication over Feedback

Although our interview guide was developed to obtain information on feedback preferences from the system to the user, participants more commonly discussed the two-way exchange of information, or communication, between the user and the AI system rather than a one-way feedback process. Participants expressed numerous preferences related to the ease, amount, invasiveness, and method of communication. In particular, easily communicating with the AI system was a priority for many people, especially in ways that would allow the user to control the system without interruption. Similarly, excess communication was viewed as burdensome, as demonstrated by the following statement: *“I think there’s a very fine line between the system asking for information and that [communication] taking you away from being productive”* (Female, London).

The amount of communication preferred by the participants varied and depended upon the amount of automation. Some participants indicated they would only want to actively communicate with the AI when information or feedback was required and did not want the system to know any information that they did not willingly input, stating, *“I don’t like things scanning things and knowing more about me than I told it*” (Female, Los Angeles). Others said they would rather have the system constantly gathering feedback or information without active user communication, resulting in full automation:

*“I think I would prefer if everything just happened for me. I don’t like messing with settings or trying to figure out what the best way to do it is. I’d rather it just dim [the lights] or change color at the right time.”* (Female, Los Angeles)

Most people were somewhere in the middle, with one participant stating, *“like if you’re driving in your car. Then, like you have control over the temperature, the AC in your car. Then, kind of comparable to that”* (Male, London). There was general agreement that the system could sense and self-adjust to enhance the environment (e.g., temperature changes). Still, the worker should have ultimate control over the AI system for other types of changes in the workspace, such as physical movement of the workstation. 

The communication topic led to discussions about ways to ensure that communication with the system would not disrupt work and flow. Participants were generally agreeable to using a calibration period when first engaging with an AI system that might require frequent prompts followed by periods of less frequent interruptions, as long as the user would have prior knowledge and control over the calibration process. As stated by one participant, *“I wouldn’t be averse to [calibration] if I just knew when it was going to happen so that I could go like, ‘Okay, it’s happening on Monday, Thursday, and then next Thursday, and I will be getting those prompts’”* (Female, London). Beyond scheduled calibration, another suggested a method to decrease disruptive communication was to combine both user input and system sensing to understand a worker’s concentration level. One participant stated,

*“I think it would be important if it had some ability to sense how focused you were at the moment because if I was in flow and it prompted me, I’d be really pissed off. I’d be like, ‘Get this thing out of my cubical immediately!’”* (Male, Los Angeles)

Participants acknowledged that measuring focus and concepts like “flow” would be difficult because work tasks vary, and productivity looks different both within and between individuals. 

**Figure 1:**
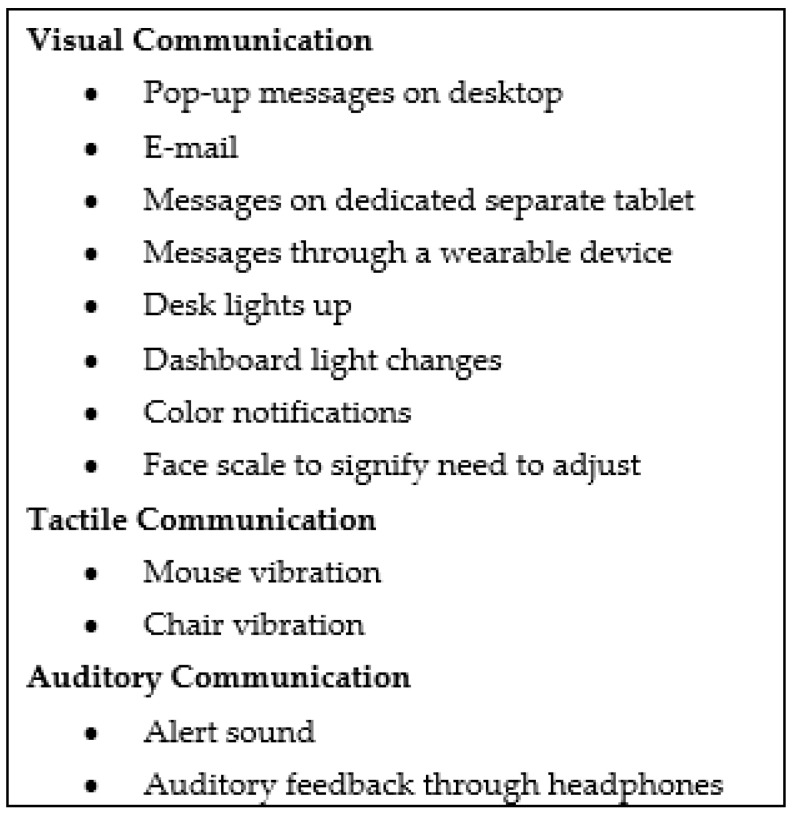
Potential methods of notification and communication by an AI workstation identified by focus group participants.

Individual preferences for a method of communication with an AI system varied greatly (Figure 1). While some participants voiced a preference for communication that is integrated with a separate device, such as a phone, tablet, or wearable device, others stated they would like all contact with an AI system to be managed through a computer on which they are working. In addition to the device used, ideas for how to best engage in communication also ranged considerably, from buzzing chairs to pop-ups on the monitor. While there was more variety in preferences than anticipated, this rich discussion into communication demonstrated the complexity behind user preferences for communication with an AI workstation. 

### 3.2. Big Data: Benefits and Perils of Monitoring

Although participants expressed a desire for fluid communication between a worker and the AI system, many also expressed concerns over the large amounts of collected data necessary for the system to learn a user’s individual needs. Specifically, several participants discussed the potential invasion of privacy and unintended uses of the data against them. In general, acceptability seemed to depend on the purpose behind data logging or tracking, as one participant noted, *“If the purpose is solely about health and well-being, definitely. But, if it is about a ‘Big Brother’ type of management … or your boss generalizes what percentage of the time you spend on what … That might be counterproductive”* (Male, London).

Participants had multiple suggestions for increasing the acceptability of data collection by an AI system. A common recommendation was to ensure de-identification of the data, along with clear transparency in how data were being logged, monitored, or used. Of note were comments indicating that data collection for research was considered more acceptable than data being obtained for business or commercial uses. As a minimum threshold, there was general agreement that outside companies should not be able to access the data, with one participant stating:

*“A written contract that the employer or whoever is guarding the data is going to reassure us that it’s going to stay with this company or stay within this administration and that it’s not going to be sold to anyone else outside of this administration.”* (Male, Los Angeles)

Some participants stated that acceptability might be increased if they could see and maintain ownership of their data. Furthermore, individuals noted that having an option to output data into an offline format that would be robust against hacking might be beneficial. One participant stated:

*“It might be a question of where the data is stored. If it could be stored somewhere where you essentially own the data and it’s not going off to some sort of big brain, or maybe you’d plug your ID pass into a computer or something, and then it reads from that. So, at the end of the day, you take that away with you, and no one else has that data but you. That might be a little more palatable for people.”* (Male, London)

There was a range of acceptability of the amount of data collected by the system. On one end of the spectrum, some participants noted that certain AI-supported functions could convince them to share as much data as needed. This sentiment is exemplified by one participant who stated, *“I personally would sign away any information you want to never overheat at my desk again”* (Male, London). This level of acceptability was in stark contrast to other participants who said they do not want any data being collected or logged for any more than immediate use by the AI system.

*“I think not recording it would be good, as in, if [the system] just gives you live updates and doesn’t store any of the data beyond five minutes. That would be really reassuring cause then they can’t, you know, do anything with five minutes; well, you could, but five minutes of data is not that much.”* (Female, London)

Finally, participants imagined some other personal uses for data collected by an AI system in the office setting. Some participants noted that having access to the accumulated data or aggregate data reports could serve as a motivator to facilitate health behavior change related to certain work productivity, stress responses, or postural outcomes that might be monitored. These discussions were compared to similar existing technologies, such as smartphone applications, smartwatches, and other wearable fitness devices. Participants noted that these data could be used as positive reinforcement, or the system could gamify those data reports to develop competition with co-workers. 

### 3.3. Work Performance and AI

Throughout all conversations related to communication and big data, work-related productivity emerged as a frequently recurring theme. Although moderators asked questions related to environmental factors and behaviors that could impact health and well-being, the interview guide did not focus on productivity; however, discussions frequently included this aspect of work performance. Participants indicated that they would like the technology to support their productivity and that communication from the technology should not interrupt their productivity in any way. Moreover, they indicated that AI should not attempt to monitor or measure productivity, particularly given the complexity of measuring work performance across different types of tasks and work contexts. Connections between productivity and well-being in terms of work performance were also raised within the focus group sessions. 

Acknowledging the complexity and diversity of office work performance, participants highlighted how an AI system could either hinder or enhance engagement. Preventing negative effects on work performance was a priority for many participants, as was the desire for an AI system to support focus and productivity. While participants were wary of too much personal data being collected, a workstation that enhances productivity was a motivating factor for allowing data collection. One participant stated, *“I would definitely be interested to know what times of day I had the highest productivity, and what I was doing for those phases because I can try to integrate that in more of my day”* (Female, Los Angeles). Participants suggested different ways of surveying the worker and sensing the worker’s behaviors for the system to understand work performance and enhance productivity. *“I think you just have to ask people. You’d have to be like, ‘Are you productive?’, set a score, then look at all the data and say, ‘These are the trends that correlate to the highest productivity’”* (Male, London).

While enhancing productivity was a desirable outcome of an AI system, participants expressed reservations about whether productivity could accurately be assessed via objective, external measures. Behavior may not be a reliable indicator, as one participant noted:

*“That is a hard problem … you mentioned earlier, ‘Oh, he stopped typing for a bit’; that means you’re thinking … which means he may be walking around, right? That seems reasonable but, on the other hand, sometimes when thinking my best thoughts, I’m not touching a computer. I’m just sort of staring into the void.”* (Male, Los Angeles)

Another participant stated similarly, *“unproductivity shows up differently in different people…cause when I’m trying to avoid my work procrastinating, I’ll just be checking my emails all day”* (Female, London). This variability in understanding and measuring productivity raised questions about whose perspective should be considered:

*“You have your own feeling, and then your boss has a feeling, and the company has a feeling … how you define a productive day. Is it that you read a few research papers and you’ve taken in the information that helps you with tasks? Or is it that you’ve done 20 tasks and therefore you’ve had a productive day?”* (Male, London).

Fundamentally, perceptions reflected the reality that work performance is most accurately understood by considering interactions among physical, cognitive, psychological, and contextual factors. Acknowledging this complexity of worker performance and productivity, participants expressed interest in understanding the relationship between performance and well-being. As summarized by one participant:

*“I would be very interested in exploring the links between health and well-being and productivity. For example, last week, a colleague of mine told me that there is a very strong direct correlation between dehydration and lack of cognitive skills…. I read a very interesting article in the Harvard Business review precisely about sitting desk vs. standing desk … Those with a sitting desk were outperformed by 50 percent by those with a standing desk. So, it suggests that there is a relationship between health and well-being and productivity.”* (Male, London).

### 3.4. Idealizations for AI in Office Workplaces

When asked to comment on a “dream workstation,” participants most frequently identified technologies and environmental modifications that could be incorporated into an AI system to optimize work performance. Of particular note, participants across all focus groups discussed ways to minimize ambient noise (e.g., quieter keyboards, warning systems for loud co-workers). Participants who hot-desk spoke of AI data logging and transfer that could support a seamless transition between workstations. There were frequent suggestions from participants that an AI system should incorporate existing technologies that support productivity (e.g., voice recognition, task tracking software) and health (e.g., fitness trackers). Similarly, many discussions related to how an AI system might use reinforcement, punitive, or mandatory processes to encourage health behaviors. One participant suggested the use of mandatory muscle stretches to gain access to the computer stating, *“because I have tendonitis, I need to [stretch]. And [I would be more compliant] if it’s forcing me to do it”* (Female, Los Angeles). While a good motivator for an individual with pain or other health conditions, others indicated mandatory solutions could be frustrating to someone without any current health condition or discomfort. Many discussions focused on the trade-offs between smart features that may support one goal but could detract from other goals. For example, if an AI-enabled system could automatically refill a cup on the desk with water, one participant immediately noted, *“then you won’t go walk to get your water”* (Female, Los Angeles).

During idealization, conversations often moved to AI systems that extended beyond the individual workstation or cubicle and instead were integrated into the building or considered larger-scale approaches. Participants frequently noted the benefits of integrated monitoring between the individual AI workstation and the building, such as knowing the building’s temperature or the amount of lighting coming from a window versus overhead lights. An individual AI workstation connected to the window blinds could automatically adjust the amount of natural light relative to overhead or workstation lights. Integration could also address concerns between individual workstations, such that sitting next to a person with opposing preferences might not nullify the individual workstations’ effects, as one person stated, *“I don’t think your desk temperature is going to make that big of a difference. You’re just going to cancel each other out, you know?”* (Female, Los Angeles). In three of the six focus groups, participants raised concern regarding negative consequences, environmental impacts, financial costs, and contextualized considerations for an AI system implemented at the scale of an entire building. One particularly poignant comment noted that efforts by an AI system to imitate nature within the indoor work environment would be wasteful when all that is necessary may be a walk outside. However, this led to debate, as other participants pointed out that not everybody has access to a safe and pleasant environment outside of the office building.

## 4. Discussion

In this study, we conducted focus group interviews to understand office workers’ perspectives on incorporating AI into the workplace as a means for improving environmental conditions and providing behavioral support to improve office worker health and well-being. Rich discussions highlighted the complexity of understanding individual needs and preferences regarding AI in office settings. There remains much speculation and caution of AI in society. Prior studies on acceptance of automation have demonstrated that safety, benefits, compatibility, privacy, and trust were common factors that influence acceptance [48,49]. These components were prevalent in our findings. We noted that the acceptability of an AI system depends on the benefits to the individual outweighing the potential detriments of a system that monitors and communicates with the user. In particular, people who were already experiencing detrimental health effects from their current work environments (i.e., tendonitis, back pain, lower cognition due to cold office temperatures) were more open to a system requiring more worker engagement. Data transparency and confidentiality, practical and concise communication, and control over the system were common desires in an AI workstation that would increase acceptability for introducing AI into office settings among the participants of this study. 

Although the moderators initially inquired about using AI to support office worker health and well-being, many groups found a connection between wellness and productivity, and many discussions of work performance emerged. These topics were particularly prevalent when participants discussed communication between the user and the AI system. In fact, participants emphasized the effect that the communication process might have on work performance, rather than on the actual mechanism through which the communication would occur. Providing communications in an effective way and well-timed within the individual work context is complex and requires careful consideration to ensure that employees are able to maintain appropriate attention to their work and feel/be productive [50]. Our findings point to a need for AI not only to sense the office environment and the position or posture of the worker but also to recognize and understand how the worker is engaging in his or her work. In addition to appropriate timing, increased communications from an AI system should be carefully considered, as the number and type of communications a worker must manage throughout a workday can directly impact overall worker well-being [51].

With projections of AI being increasingly used as autonomous support systems in the workplace [35], there is a need to understand how incorporating an AI system may affect work behavior. Machine learning has been applied to sense human activity through wearable accelerometers to computer vision techniques [52,53]. However, office work behavior is complex, and the process for understanding work performance through sensing has yet to be developed or validated. As a foundation, it is necessary to analyze office worker behavior to know how an AI system might effectively sense worker engagement to support office work performance. Rather than viewing the person and their environment as separate entities, a transactional perspective that focuses on the interplays among the worker, technologies, and workplace environment would be a useful approach for developing such workplace AI solutions [54]. This type of approach would be beneficial in the ongoing training of an AI system that must learn and change based on the user to achieve the ultimate goal of altering a worker’s behaviors [55]. 

In addition to the benefits to worker health and well-being, developing effective automation within office environments may have broader organizational and environmental benefits. Of importance at an organizational level are opportunities to improve the financial bottom-line due to increased worker satisfaction and well-being and decreased absenteeism or presenteeism, all of which can lead to increased performance and productivity [56]. In particular, automation has been shown to save energy and lower operating costs [57,58]. Participants in our focus groups briefly mentioned ways in which AI incorporated at individual workstations may be able to connect to adjustments at the building level that could benefit all workers and energy costs (e.g., automated adjustment of window coverings). In addition, AI systems integrated into individual workstations could turn off lights, personal comfort devices, and other electronic devices when the presence of the worker is not noted as a means of conserving energy [42]. Future study is needed to explore the transactions that occur among a worker, the workspace, and an AI system to better understand these interplays and the implications of worker health, work performance, and other organizational or environmental impacts within future office work contexts [26]. Such a multifaceted approach could result in robust, efficacious workplace worker health solutions [20,34] with additional far-reaching benefits.

Findings of this study should be considered with multiple limitations in mind. These focus groups were conducted as part of a broader study examining the development of an AI-enabled office workstation to enhance office worker health and productivity. While open discussions were encouraged, focus group questions were framed around relevant topics of interest in the broader AI development project, which may have limited participants from suggesting ideas or discussing issues that diverged from the study’s focus. Although we did compare discussions by the employer and geographic location, we did not collect detailed demographic information of our participants beyond their sex. As such, we were not able to conduct any robust analysis of other individual worker characteristics (e.g., age, health status). To minimize potential respondent bias, no individuals with knowledge of the specific research study aims or individuals with a direct working relationship to the moderators were included in the focus groups. However, due to network sampling, many participants worked in jobs that involved research and engineering, potentially making them more open to new technologies than other contemporary office workers. It is not clear if users less familiar with AI solutions or working in office settings that are not accustomed to research and development would share these perspectives and preferences. Moreover, the specific findings reported by these workers may not translate to workers in other countries, regions, or cultures.

## 5. Conclusions

In this study, we explored office worker preferences for incorporating AI into the office workspace. Our findings indicate there is a wide range of individual preferences and areas of concern. While likely beneficial for improving healthy and productive engagement in the workplace, rich discussions among office workers demonstrated how the acceptability of AI within an office setting is complex and affected by the person, the work, and the organizational context. The key components that emerged from focus group interviews were preferences around data security, communication with the system, and impact on work performance and productivity. Future studies should explore trust in technology and work performance related to AI systems to offer further insight into designing AI systems within office settings to support work performance and worker well-being.

## Data Availability

The data presented in this study are available on request from the corresponding author. The data are not publicly available due to potential privacy concerns that may arise based on conversations held within the focus group sessions. For individuals wishing to conduct further analysis, access to transcripts is available through a data sharing agreement approved by the relevant intuitional review boards.

## Appendix A

**Focus Group Interview Guide.**

**Introduction, Moderator Statement:** We are looking at opportunities around making the work environment healthier for people. For example, there might be an artificial intelligence (AI) system that asks you to do healthier things like change the office temperature, change the room lighting, improve your posture, or change your position from sit to stand. We will use this data to inform the development of such technology. We ask you not share anything that is said as part of this focus group outside the room. To start things off, could you each please tell us a little bit about yourself, your role at [Arup, USC], and what your office set-up looks like.

**Feedback Options, Moderator Statement:** Imagine there was AI in your office workspace. This AI could help manage things in your office to help you be healthier and more comfortable. It might change the office temperature, change the room lighting, ask you to change your posture or position at the desk. How might you like the AI to provide feedback to you or ask you about these changes?

Follow-up prompts:

- If not mentioned or respondents need examples, ideas mention: phone notification, pop-up on the computer screen; vibration/buzzer on the desk; blinking lights on the desk; notifications on a different dedicated screen placed on the desk (like a tablet); a virtual agent who you can talk to that might appear on multiple mediums (e.g., computer monitor, phone, computer, tablet).
- Regarding changes to the environment (e.g., lighting, temperature, noise) would you prefer that the system ask or automatically adjust the environment based on what it thinks you need or prefer without requesting feedback? For example, turning on a fan when you might be too warm, turning on a space heater when you might be too cold, adjusting the lights or monitor brightness when you are squinting or there is glare from an outside source;
- Regarding posture or position changes, would you prefer feedback or for the AI to automatically make changes to the workstation? For example, raising the desk when you’ve been sitting too long, raising or tilting the monitor when you are looking up or there is glare from an outside source;
- Which feedback options that we’ve talked about do you like the best? Least? Why?
- Are there any additional problems you can think of with the feedback options? Additional benefits?

**Sensing Options, Moderator Statement:** To be able to make intelligent suggestions regarding the environment (e.g., lighting, temperature, noise) and your behaviors (e.g., posture, positioning), the AI would also need to sense things about your health, like your body temperature or muscle tension, and the way in which you are positioned or engaging at your workstation. We would like to hear your thoughts about how and AI system might obtain these data, consider sensors that you might wear or that might be placed at the workstation to monitor your health. What kinds of sensors that you know of would you feel most comfortable with?

Follow-up prompts:

- If not mentioned or respondents need examples, ideas include: a smart watch or fitness tracker; thermal cameras on the desk (no pictures; no identifiable info); Kinect camera or webcam to identify postures or activities; muscle activation sensors (e.g., EMG electrodes); light sensors; occupancy sensors to detect if the desk is used or not; sensors on peripheral devices connected to the desk or workstation;
- Which of these sensor options that we’ve talked about do you like the best? Least? Why?
- Are there any additional problems you can think of with the sensor options? Additional benefits?

**Other Features and Capabilities, Moderator Statement:** An AI system in your workspace could also have additional features and capabilities that go beyond adjusting the environment or promoting healthy posture or positioning. If it could include anything you might image, we would like to know what kinds of features or capabilities you would like for an AI system to have. We are most interested in ways in which an AI system could address problems and unmet needs in your work environment. What other problems or unmet needs can you think of in your work environment, and what kinds of features or capabilities in an AI system might help?

Follow-up prompts:

- If not mentioned or respondents need examples, ideas include: appointment, meeting, or important deadline reminders; acts as a personal assistant (e.g., orders lunch, makes reservations, auto-replies to e-mail requests); plays your favorite music to cheer you up you are down; playing calming music when you are stressed;
- Which of the features or capabilities that we’ve talked about do you like the best? Least? Why?
- Are there any additional problems you can think of with these features? Additional benefits?

**Conclusion, Moderator Statement:** We have talked about a lot of ideas related to how an AI system could be incorporated in your office workstation to promote health and comfort, including options for the system to provide feedback, ways such a system could sense your health or needs within the environment, and your ideas for other things that you might want an AI system to do. Does anyone have any final comments or ideas across any of these areas before we conclude?

## References

[1] Clausen G., Wyon D.P. (2008). The combined effects of many different indoor environmental factors on acceptability and office work performance. HVAC R Res..

[2] de Croon E.M., Sluiter J.K., Kuijer P.P.F.M., Frings-Dresen M.H.W. (2005). The effect of office concepts on worker health and performance: A systematic review of the literature. Ergonomics.

[3] Wong S.K., Lai L.W.C., Ho D.C.W., Chau K.W., Lam C.L.K., Ng C.H.F. (2009). Sick building syndrome and perceived indoor environmental quality: A survey of apartment buildings in Hong Kong. Habitat Int..

[4] van Marken Lichtenbelt W., Hanssen M., Pallubinsky H., Kingma B., Schellen L. (2017). Healthy excursions outside the thermal comfort zone. Build. Res. Inf..

[5] Hedge A., Miller L., Dorsey J.A. (2014). Occupant comfort and health in green and conventional university buildings. Work.

[6] van Bommel W., van den Beld G. (2004). Lighting for work: A review of visual and biological effects. Light. Res. Technol..

[7] Boyce P.R.B. (2010). The impact of light in buildings on human health. Indoor Built Environ..

[8] Heydarian A., Pantazis E., Carneiro J.P., Gerber D., Becerik-Gerber B. (2016). Lights, building, action: Impact of default lighting settings on occupant behaviour. J. Environ. Psychol..

[9] Mak C., Lui Y. (2012). The effect of sound on office productivity. Build. Serv. Eng. Res. Technol..

[10] Di Blasio S., Shtrepi L., Puglisi G.E., Astolfi A. (2019). A cross-sectional survey on the impact of irrelevant speech noise on annoyance, mental health and well-being, performance and occupants’ behavior in shared and open-plan offices. Int. J. Environ. Res. Public Health.

[11] Joines S., James T., Liu S., Wang W., Dunn R., Cohen S. (2015). Adjustable task lighting: Field study assesses the benefits in an office environment. Work.

[12] Janwantanakul P., Pensri P., Jiamjarasrangsri V., Sinsongsook T. (2008). Prevalence of self-reported musculoskeletal symptoms among office workers. Occup. Med..

[13] Soriano A., Kozusznik M., Peiró J. (2018). From Office environmental stressors to work performance: The role of work patterns. Int. J. Environ. Res. Public Health.

[14] Tudor-Locke C., Leonardi C., Johnson W.D., Katzmarzyk P.T. (2011). Time spent in physical activity and sedentary behaviors on the working day: The American Time Use Survey. J. Occup. Environ. Med..

[15] Biswas A., Oh P.I., Faulkner G.E., Bajaj R.R., Silver M.A., Mitchell M.S., Alter D.A. (2015). Sedentary time and its association with risk for disease incidence, mortality, and hospitalization in adults a systematic review and meta-analysis. Ann. Intern. Med..

[16] De Rezende L.F.M., Rey-López J.P., Matsudo V.K.R., Luiz O.D.C. (2014). Sedentary behavior and health outcomes among older adults: A systematic review. BMC Public Health.

[17] Edwardson C.L., Gorely T., Davies M.J., Gray L.J., Khunti K., Wilmot E.G., Yates T., Biddle S.J.H. (2012). Association of sedentary behaviour with metabolic syndrome: A meta-analysis. PLoS ONE.

[18] Falck R.S., Davis J.C., Liu-Ambrose T. (2017). What is the association between sedentary behaviour and cognitive function? A systematic review. Br. J. Sports Med..

[19] Baker R., Coenen P., Howie E., Williamson A., Straker L. (2018). The short term musculoskeletal and cognitive effects of prolonged sitting during office computer work. Int. J. Environ. Res. Public Health.

[20] Tamers S.L., Streit J., Pana-Cryan R., Ray T., Syron L., Flynn M.A., Castillo D., Roth G., Geraci C., Guerin R. (2020). Envisioning the future of work to safeguard the safety, health, and well-being of the workforce: A perspective from the CDC’s National Institute for Occupational Safety and Health. Am. J. Ind. Med..

[21] Millward L.J., Haslam S.A., Postmes T. (2007). Putting employees in their place: The impact of hot desking on organizational and team identification. Organ. Sci..

[22] Cooper P.B., Maraslis K., Tryfonas T., Oikonomou G. (2017). An intelligent hot-desking model harnessing the power of occupancy sensing data. Facilities.

[23] Parker L.D. (2020). The COVID-19 office in transition: Cost, efficiency and the social responsibility business case. Account. Audit. Account. J..

[24] Green N., Tappin D., Bentley T. (2020). Working From Home Before, During and After the Covid-19 Pandemic: Implications for Workers and Organisations. N. Z. J. Employ. Relat..

[25] Xiao Y., Becerik-Gerber B., Lucas G., Roll S.C. (2020). Impacts of working from home during COVID-19 pandemic on physical and mental well-being of office workstation users. J. Occup. Environ. Med..

[26] Felknor S., Streit J., Chosewood L., McDaniel M., Schulte P., Delclos G. (2020). How will the future of work shape the OSH professional of the future? A workshop summary. Int. J. Environ. Res. Public Health.

[27] Pereira M.J., Johnston V., Straker L.M., Sjøgaard G., Melloh M., O’Leary S.P., Comans T.A. (2017). An investigation of self-reported health-related productivity loss in office workers and associations with individual and work-related factors using an employer’s perspective. J. Occup. Environ. Med..

[28] Shrestha N., Kukkonen-Harjula K.T., Verbeek J.H., Ijaz S., Hermans V., Bhaumik S. (2018). Workplace interventions for reducing sitting at work. Cochrane Database Syst. Rev..

[29] Parry S.P., Coenen P., Shrestha N., O’Sullivan P.B., Maher C.G., Straker L.M. (2019). Workplace interventions for increasing standing or walking for decreasing musculoskeletal symptoms in sedentary workers. Cochrane Database Syst. Rev..

[30] Song Z., Baicker K. (2019). Effect of a workplace wellness program on employee health and economic outcomes: A randomized clinical trial. JAMA J. Am. Med. Assoc..

[31] Muir S.D., Silva S.S.M., Woldegiorgis M.A., Rider H., Meyer D., Jayawardana M.W. (2019). Predictors of success of workplace physical activity interventions: A systematic review. J. Phys. Act. Heal..

[32] Lecours A. (2020). Using an occupational perspective to understand behaviours fostering the prevention of work-related health problems: A proposed conceptual model. J. Occup. Sci..

[33] Clark F., Sanders K., Carlson M., Blanche E., Jackson J. (2007). Synthesis of habit theory. OTJR Occup. Particip. Health.

[34] Chari R., Chang C.C., Sauter S.L., Sayers E.L.P., Cerully J.L., Schulte P., Schill A.L., Uscher-Pines L. (2018). Expanding the paradigm of occupational safety and health: A new framework for worker well-being. J. Occup. Environ. Med..

[35] Wisskirchen G., Biacabe B.T., Bormann U., Muntz A., Niehaus G., Soler G.J., Von Brauchitsch B. (2017). Artificial Intelligence and Robotics and Their Impact on the Workplace.

[36] Manuvinakurike R., Velicer W.F., Bickmore T.W. (2014). Automated indexing of Internet stories for health behavior change: Weight loss attitude pilot study. J. Med. Internet Res..

[37] Wang Y., Khooshabeh P., Gratch J. (2013). Looking real and making mistakes. Proceedings of the Lecture Notes in Computer Science (Including Subseries Lecture Notes in Artificial Intelligence and Lecture Notes in Bioinformatics).

[38] Blascovich J., McCall C., Dill K.E. (2013). Social Influence in Virtual Environments. Oxford Handbook of Media Psychology.

[39] Traum D., Swartout W., Marsella S., Gratch J. (2005). Fight, flight, or negotiate: Believable strategies for conversing under crisis. Proceedings of the Lecture Notes in Computer Science (Including Subseries Lecture Notes in Artificial Intelligence and Lecture Notes in Bioinformatics).

[40] Ernst E., Merola R., Samaan D. (2019). Economics of artificial intelligence: Implications for the future of work. IZA J. Labor Policy.

[41] Buchanan B.G. (2005). A (Very) Brief History of Artificial Intelligence. AI Mag..

[42] Aryal A., Becerik-Gerber B., Anselmo F., Roll S.C., Lucas G.M. (2019). Smart desks to promote comfort, health, and productivity in offices: A vision for future workplaces. Front. Built Environ..

[43] Hammarberg K., Kirkman M., De Lacey S. (2016). Qualitative research methods: When to use them and how to judge them. Hum. Reprod..

[44] Kamberelis G., Dimitriadis G. (2011). Focus groups: Contingent articulations of pedagogy, politics, and inquiry. The Sage handbook of qualitative research. SAGE Handb. Qual. Res..

[45] Kamberelis G., Dimitriadis G. (2013). Focus Groups.

[46] Malterud K. (2012). Systematic text condensation: A strategy for qualitative analysis. Scand. J. Public Health.

[47] Maykut P. (2002). Beginning Qualitative Research.

[48] Motamedi S., Wang P., Zhang T., Chan C.Y. (2020). Acceptance of full driving automation: Personally owned and shared-use concepts. Hum. Factors.

[49] Lau J., Zimmerman B., Schaub F. (2018). Alexa, are you listening? Privacy perceptions, concerns and privacy-seeking behaviors with smart speakers. Proc. ACM Hum. Comput. Interact..

[50] Mark G., Iqbal S., Czerwinski M., Johns P. (2015). Focused, aroused, but so distractible: A temporal perspective on multitasking and communications. Proceedings of the 2015 ACM International Conference on Computer-Supported Cooperative Work and Social Computing.

[51] Bordi L., Okkonen J., Mäkiniemi J.P., Heikkilä-Tammi K. (2018). Communication in the digital work environment: Implications for wellbeing at work. Nord. J. Work. Life Stud..

[52] Cha S.H., Seo J., Baek S.H., Koo C. (2018). Towards a well-planned, activity-based work environment: Automated recognition of office activities using accelerometers. Build. Environ..

[53] Vrigkas M., Nikou C., Kakadiaris I.A. (2015). A review of human activity recognition methods. Front. Robot. AI.

[54] Dickie V., Cutchin M.P., Humphry R. (2006). Occupation as transactional experience: A critique of individualism in occupational science. J. Occup. Sci..

[55] Aldrich R.M. (2008). From complexity theory to transactionalism: Moving occupational science forward in theorizing the complexities of behavior. J. Occup. Sci..

[56] Nielsen K., Nielsen M.B., Ogbonnaya C., Känsälä M., Saari E., Isaksson K. (2017). Workplace resources to improve both employee well-being and performance: A systematic review and meta-analysis. Work Stress.

[57] Tientcheu S.I.N., Chowdhury S.P., Olwal T.O. (2019). Intelligent energy management strategy for automated office buildings. Energies.

[58] Von Neida B., Maniccia D., Tweed A. (2001). An analysis of the energy and cost savings potential of occupancy sensors for commercial lighting systems. J. Illum. Eng. Soc..

